# Impact of prior antibiotics on infected pancreatic necrosis microbiology in ICU patients: a retrospective cohort study

**DOI:** 10.1186/s13613-020-00698-0

**Published:** 2020-06-15

**Authors:** Charlotte Garret, Emmanuel Canet, Stéphane Corvec, David Boutoille, Matthieu Péron, Isabelle Archambeaud, Aurélie Le Thuaut, Jean-Baptiste Lascarrou, Frédéric Douane, Marc Lerhun, Nicolas Regenet, Emmanuel Coron, Jean Reignier

**Affiliations:** 1 Service de Médecine Intensive et Réanimation, Centre Hospitalier Universitaire de Nantes, 1 Place Alexis Ricordeau, 44093 Nantes, France; 2 Service de Bactériologie-Hygiène Hospitalière, CHU de Nantes, 1 Place Alexis Ricordeau, 44093 Nantes, France; 3 CRCINA, INSERM U1232, Université de Nantes, Nantes, France; 4 Service de Maladies Infectieuses et Tropicales, CHU de Nantes, 1 Place Alexis Ricordeau, 44093 Nantes, France; 5 Institut des Maladies de l’appareil Digestif, CHU de Nantes, 1 Place Alexis Ricordeau, 44093 Nantes, France; 6 Département de Biostatistiques, CHU de Nantes, 1 Place Alexis Ricordeau, 44093 Nantes, France

**Keywords:** Acute pancreatitis, Infected pancreatic necrosis, Multidrug-resistant infection, Step-up approach

## Abstract

**Background:**

Recent guidelines advise against prophylactic antibiotics in patients with necrotizing pancreatitis, advocating instead a step-up drainage and necrosectomy strategy with antibiotics as dictated by microbiological findings. However, prompt antibiotic therapy is recommended in patients with sepsis or septic shock, a possible presentation of infected pancreatic necrosis (IPN). Consequently, in many critically ill patients with IPN, pancreatic samples are collected only after broad-spectrum antibiotic therapy initiation. Whether this prior antibiotic exposure alters the microbiological findings is unknown. The main objective was to determine whether prior antibiotic exposure sterilized the samples collected during procedures for suspected IPN in patients admitted to the intensive care unit (ICU) for acute pancreatitis with suspected IPN. We retrospectively studied 56 consecutive ICU patients admitted with suspected IPN. We collected details on the microbiological samples and antimicrobials used. A definite diagnosis of IPN was given when bacteria were identified in pancreatic samples.

**Results:**

In all, 137 pancreatic samples were collected, including 91 (66.4%) after antibiotic therapy initiation. IPN was confirmed in 48 (86%) patients. The proportion of positive samples was 74 (81.3%) in antibiotic-exposed patients and 32/46 (69.5%) in unexposed patients (*p *= 0.58). Of the 74 positive samples from exposed patients, 62 (84%) had organisms susceptible to the antibiotics used. One-third of samples contained more than one organism. Among patients with IPN, 37.5% had positive blood cultures. Multidrug- or extensively drug-resistant bacteria were identified at some point in half the patients. *Enterobacter cloacae* complex was more frequent in the exposed group (*p* = 0.02), as were Gram-negative anaerobic bacteria (*p* = 0.03).

**Conclusion:**

Antibiotic exposure before sampling did not seem to affect culture positivity of pancreatic samples to confirm IPN, but may affect microbiological findings. Our results suggest that, in patients with sepsis and suspected IPN, antibiotics should be started immediately and pancreatic samples obtained as soon as possible thereafter. In other situations, antibiotics can be withheld until the microbiological results of pancreatic samples are available, to ensure accurate targeting of the spectrum to bacterial susceptibility patterns.

*ClinicalTrials.gov number* NCT03253861

## Background

Severe acute pancreatitis is a common reason for intensive care unit (ICU) admission and is associated with long hospital stays and high morbidity and mortality rates [1, 2]. Infected pancreatic necrosis (IPN) develops in about one-third of patients, who are then at increased risk for death [3]. International guidelines about the management of IPN [4–8] recommend a step-up strategy, based on findings from randomized controlled trials [9, 10]. The first step is minimally invasive (percutaneous or endoscopic) drainage combined with broad-spectrum antibiotic therapy. When this fails, minimally invasive endoscopic or surgical necrosectomy is performed. Microbiological studies are performed on all drainage and necrosectomy samples.

Prophylactic antibiotic therapy was not helpful in preventing IPN in several randomized controlled trials, and is therefore not recommended [11–13]. However, most international guidelines [4, 5, 14, 15] fail to provide recommendations about using antibiotics in patients with IPN, perhaps due to the scarcity of relevant published data. Canadian guidelines [16] recommend reserving antibiotics for patients with confirmed IPN and tailoring the antibiotic regimen to the species and sensitivities of the bacteria recovered from necrotic tissue samples. However, these guidelines also suggest that empirical antibiotic therapy may be considered before the culture results are available.

For sepsis and septic shock, in contrast, there is a clear recommendation to start antibiotics as early as possible [17]. Because sepsis and septic shock are possible presentations of IPN, many patients with IPN therefore receive antibiotics before the diagnosis of IPN is made or before a pancreatic sample can be collected. Furthermore, patients with IPN may require immediate antibiotic therapy for a co-existing infection at another site. The penetration of antibiotics within foci of pancreatic necrosis has been poorly evaluated but may be limited [18–26]. Thus, whether antibiotic exposure before the collection of pancreatic samples sterilizes the microbiological findings is unclear. Importantly, studies have documented a rise in the proportion of patients with pancreatic samples positive for multidrug-resistant bacteria (MDRB) up to values of 52% [27] and 63% [28]. Limiting the development of bacterial resistance by restricting the use of antibiotics is clearly a major public health goal [29–31]. If antibiotic exposure before pancreatic sampling hinders the identification of causative organisms, then exposed patients might not only have poorer outcomes, but also require longer and/or broader antibiotic treatments at higher risk for selecting resistant strains, and negative samples may not be sufficient reason to stop the antibiotics.

Our primary objective was to investigate whether the microbiology of pancreatic samples from critically ill patients with suspected IPN was sterilized according to whether antibiotics were started before or only after sample collection. The secondary objective was to describe the bacterial species recovered and their resistance patterns in the groups previously exposed vs. unexposed to antibiotics.

## Methods

### Study design and setting

We performed a retrospective cohort study in consecutive patients admitted from 1 January 2012 to 2031 December 2015 to the ICU of the Hôtel-Dieu University Hospital, Nantes, France, for acute pancreatitis with suspected IPN. The study data were extracted from the electronic health records of each patient (Millennium database, Nantes, France).

The ethics committee of the French Intensive Care Society approved the study (#CE SRLF16-09). In accordance with French law on retrospective studies of anonymized data, informed consent was not required.

### Patient eligibility criteria and definitions

Critically ill adults (≥ 18 years) admitted to our ICU during the study period for acute pancreatitis (ICD-10 codes K85.0 to K85.9) were identified by searching the hospital electronic database. The electronic health records of all identified patients were reviewed by one of us (CG). According to the Atlanta classification [32], acute pancreatitis was classified as moderately severe in patients with transient organ failure resolving within 48 h and/or local or systemic complications without persistent organ failure and as severe in patients with organ failure persisting for more than 48 h. Organ failure was defined as a modified Marshall score (33) ≥ 2 for the renal, respiratory, and/or cardiovascular system (Additional file 1). Among patients with moderately severe or severe acute pancreatitis, those who had suspected IPN were identified. Suspected IPN was defined as computed tomography (CT) evidence of pancreatic or peripancreatic necrosis and performance of an invasive pancreatic procedure (percutaneous or endoscopic fluid drainage and/or endoscopic or surgical necrosectomy) [9, 10]. Patients with suspected IPN and available data on the microbiology of the pancreatic samples were included in the study.

Definite IPN was defined as either CT evidence of a collection containing extraluminal gas or a positive culture of pancreatic tissue obtained by drainage or necrosectomy [9]. Possible IPN was defined as pancreatic and/or peripancreatic necrosis requiring drainage/necrosectomy, persistent sepsis, and negative cultures of the pancreatic samples, in the absence of infection at another site [7].

### Data collection and outcomes

For each patient, we collected demographics, comorbidities, CT findings including the CT Severity Index (CTSI) calculated by a radiologist, pancreatic procedures (percutaneous or endoscopic transluminal drainage and endoscopic or surgical necrosectomy), and the treatments used in the ICU including antibiotics. At our institution, intensivists, gastroenterologists, and digestive surgeons are available 24 h a day, 7 days a week and work together to manage patients with acute pancreatitis. For patients with IPN, our local policy is to apply the step-up approach described by van Santvoort et al. [9]. Selection of the antibiotics and the duration of antibiotic therapy are at the discretion of the attending physician. We recorded the following complications: hollow organ perforation requiring a surgical, radiological, or endoscopic procedure; bowel ischemia confirmed by CT, endoscopy, or surgery; abdominal and/or gastrointestinal bleeding requiring a surgical, radiological, or endoscopic procedure, and gastrointestinal bleeding with loss of more than 500 mL of blood/24 h. We also collected ICU and hospital mortality. The microbiological data were recorded in detail, with the number and nature of the samples, collection method, culture results, and antimicrobial susceptibility test findings. Each sample indicates a separate procedure. Bacteria were classified into four categories according to their antibiotic susceptibility profile [33]: bacteria without acquired antimicrobial resistance, multidrug-resistant bacteria (MDRB, defined as acquired non-susceptibility to at least one agent in three or more antimicrobial categories); extensively drug-resistant bacteria (XDRB, defined as non-susceptibility to at least one agent in all but two or fewer antimicrobial categories); and pandrug-resistant bacteria (defined as resistant to all agents in all antimicrobial categories). We compared patients who were started on antibiotics more than 24 h before the first pancreatic sample was collected (exposed group) to the other patients (unexposed group).

### Statistical analysis

Quantitative variables were described as median and interquartile range [IQR] and compared using the Mann–Whitney tests, whereas qualitative variables were described as number (%) and compared using Fisher’s test. All tests were two-sided and *p* values lower than 5% were considered to indicate significant associations. Statistical tests were conducted using the SAS 5.0.1 software package (SAS Institute Inc., Cary, NC).

## Results

### Study population

**Fig. 1:**
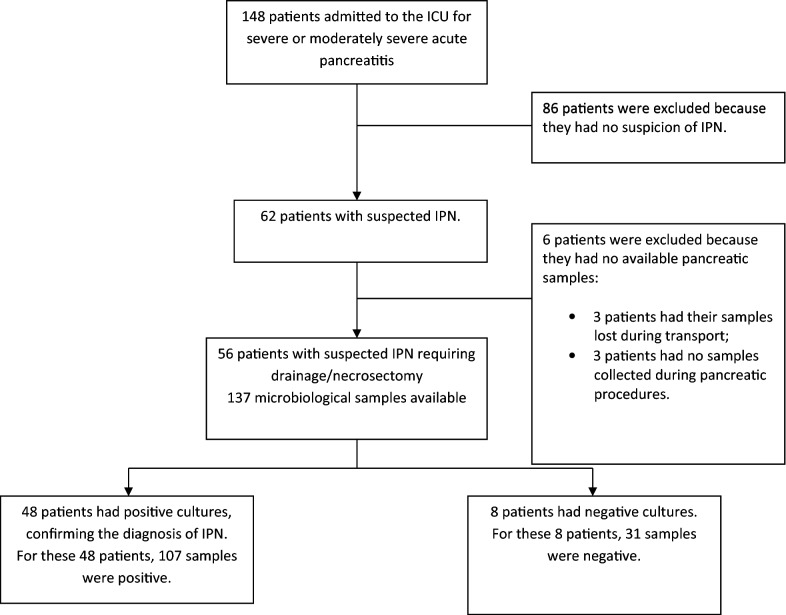
Patient flowchart

Figure 1 is the patient flowchart. Of 148 patients admitted for moderately severe or severe acute pancreatitis, 56 had suspected IPN and were included. Table 1 reports their main characteristics. Of the 56 patients, 33 (59%) were and 23 were not started on antibiotics more than 24 h before collection of the first pancreatic sample. No significant differences in baseline features or outcomes were found between the exposed and unexposed groups (Table 1). CT showed gas bubbles within necrotic collections in 14 (25%) patients. The median number of pancreatic samples per patient was 2 [1–3] and the total number of samples in the 56 patients was 137. The sample cultures were positive in 48 (85.7%) patients, and 8 patients (14.3%), IPN was considered as possible. Of these 48 patients with a definite diagnosis of IPN, 18 (37.5%) had positive blood cultures, with the same bacterial species as in the pancreatic samples.

**Table 1:** Baseline features of the study patients overall and in the antibiotic-exposed and unexposed groups^a^

	Suspected IPN, *n* = 56	Exposed, *n* = 33	Unexposed, *n* = 23	*P* value
Age, y, median [IQR]	58 [45.5;67.5]	54.5 [45.5;64]	63 [45;68.5]	0.89
Males, *n* (%)	47 [84]	26 [78]	21 [91]	0.28
BMI, median [IQR]	26.3 [23.6;29.5]	25.7 [23, 29]	26.9 [24.9;33]	0.18
Origin of pancreatitis				
Biliary	16 (29)	9 (27)	7 (30.5)	0.95
Alcohol abuse	23 (51)	14 (43)	9 (39)	
Other^b^	17 (30)	10 (30)	7 (30.5)	
SAPS II^c^, median [IQR]	38 (28;52)	37 [24;52]	39 [31;52]	0.73
CTSI^d^, median [IQR]	6 [4, 9]	6 [4, 9]	6 [4, 9]	0.79
No organ failure^e^, *n* (%)	1 (2)	0	1 (5)	0.76
1 or 2 organ failures, *n* (%)	41 (73)	24 (73)	17 (74)	
≥3 organ failures, *n* (%)	14 (25)	9 (27)	5 (21)	
Type of organ failure, *n* (%)				
Respiratory failure	52 (93)	31 (94)	21 (92)	0.99
Mechanical ventilation	43 (77)	24 (73)	19 (83)	0.52
Circulatory failure	34 (61)	20 (61)	14 (61)	0.99
Renal failure	33 (59)	18 (55)	15 (65)	0.58
Time from ICU admission to first procedure for IPN, days, median [IQR]	21 [12, 29]	25 [15, 36]	19 [10–25]	0.61
IPN outcomes				
Total number of procedures for IPN, median [IQR]	2 [1–3]	2 [1.5–3]	2 [1–2.75]	0.99
Confirmed IPN (positive sample culture), *n (%)*	48 (85.7)	30 (90)	18 (78)	0.25
Number of positive samples/total number of samples (%)	106 (77)	74/91 (81)	32/46 (70)	0.58
Patients developing a multidrug-resistant infection^f^, *n (%)*	15 (27)	8 (24)	6 (26)	0.99
Patients developing an extensively drug resistant infection^g^, *n (%)*	10 (17.8)	6 (18)	4 (17)	0.99
Patients with concomitant positive blood culture, *n (%)*	18 (32)	8 (24)	10 (43)	0.15
Other outcomes				
Perforation of hollow organ, *n (%)*	5 (9)	4 (12)	1 (4)	0.63
Bowel ischemia, *n (%)*	3 (5)	2 (6)	1 (4)	0.99
Intestinal bleeding, *n (%)*	11 (20)	5 (15)	6 (26)	0.33
ICU stay, days, median [IQR]	24.5 [7–47]	23 [8–48]	28 [6–44]	0.54
Hospital stay, days, median [IQR]	66.5 [42–96]	68 [46.5–96]	63 [42–101]	0.68
ICU mortality, *n (%)*	9 (16)	5 (15)	4 (17)	0.99
Hospital mortality, *n (%)*	9 (16)	5 (15)	4 (17)	0.99

*IPN* infected pancreatic necrosis, *IQR* interquartile range, *BMI* body mass index, *SAPS II* Simplified Acute Physiology Score version II, *CTSI* Computed Tomography Severity Index, *ICU* intensive care unit

^a^Exposure was defined as patients who were started on antibiotics more than 24 h before the first pancreatic sample was collected

^b^Other causes of pancreatitis: hypertriglyceridemia, drugs, endoscopic retrograde cholangiopancreatography, and unknown

^c^The SAPS II can range from 0 (least severe) to 163 (most severe, with a 100% predicted risk of death); patients with a score of 50 have a 46.1% predicted risk of death

^d^The CTSI can range from 0 to 10; the predicted risk of death is 6% for values in the 4–6 range and 17% for values in the 7–10 range

^e^Organ failure was defined as a modified Marshall score (Supplemental Digital Content 1) ≥ 2 for the renal, respiratory, or cardiovascular system

^f^Multidrug-resistant bacteria were defined as bacteria with acquired non-susceptibility to at least one agent in three or more antimicrobial categories

^g^Extensively drug-resistant bacteria were defined as non-susceptibility to at least one agent in all but two or fewer antimicrobial categories

### Antimicrobial therapy and culture positivity in the exposed and unexposed groups

In the 33 patients in the exposed group, the reasons for previous antibiotic exposure were suspected IPN without shock (*n* = 10, 30%), suspected IPN with septic shock (*n* = 10, 30%), and extra-abdominal infection (*n* = 13, 39%; 6 for pneumonia, 3 for catheter-related infection, and 1 for urinary tract infection). The median time on antibiotics before sampling was 7 [3–10] days. The antibiotics used before sampling were broad-spectrum penicillins in half the patients (Table 2). The median duration of overall antimicrobial therapy in the ICU was 47 [28–77] days, and the number of antibiotics received overall in the ICU was significantly higher in the exposed group (5 [4–8] days versus 3 [2–5] days, *p* = 0.002).Of the 137 samples, 91 (66.4%) were from exposed patients. Of the 91 samples from exposed patients, 74 (81.3%) were positive, and of the 41 samples from unexposed patients, 32 (78%) were positive. The proportion of positive cultures was not significantly different between the exposed and unexposed groups (Table 1).

**Table 2:** Characteristics of antimicrobial therapy: main molecule used and duration

	Total patients *n* = 56	Exposed group^a^*n* = 33	Unexposed group *n* = 23	*P* value
First line antimicrobial therapy, *n* (%)				
Penicillin (amoxicillin and Peni M)	6 (11)	2 (6)	4 (17)	0.4
Amoxicillin–clavulanic acid	10 (18)	4 (12)	6 (26)	0.3
Piperacillin–tazobactam	16 (29)	9 (27)	7 (30)	1
Carbapenem	11 (19)	10 (30)	1 (4)	0.08
Cephalosporin	11 (19)	7 (21)	4 (17)	1
Fluoroquinolone	1 (2)	1 (3)	0	1
Cotrimoxazole	1 (2)	0	1 (4)	0.42
Metronidazole	33 (59)	17 (52)	16 (70)	0.51
Aminoglycoside	11 (19)	7 (21)	4 (17)	1
Anti-fungal	6 (11)	4 (12)	2 (9)	1
Time of overall antimicrobial therapy in ICU (median in days [IQR])	47 [28–77]	50 [28–88]	38.5 [26–61]	0.18
Number of overall different antimicrobial agents in ICU (median [IQR])	4 [2–6]	5 [4–8]	3 [2–5]	*0.002*

Italic value indicates significance of *P* value (*P* < 0.05)

^a^Exposure was defined as patients who were started on antibiotics more than 24 h before the first pancreatic sample was collected

Of the 74 positive samples from exposed patients, 62 were positive for bacteria showing in vitro susceptibility to the pre-sampling antibiotics. Of these 62 samples, 30 were collected after more than 24 h, but less than 7 days on antibiotics and 32 after more than 7 days on antibiotics (Table 3).

**Table 3:** Distribution of microorganisms recovered at the time of the first sampling in 107 positive samples in the groups exposed and unexposed to antibiotics

	Total patients with confirmed IPN *n* = 48 Species *n* = 162 (100%)	Exposed group *n* = 30 species *n* = 97 (60%)	Unexposed group *n* = 18 species *n *=65 (40%)	*P* value
Bacteria				
Gram-negative enterobacteriaceae	*82 (50.6)*	*49 (50.5)*	*33 (50.5)*	*1*
*Escherichia coli*	36 (22.2)	16	20	
*Klebsiella pneumoniae*	11 (6.8)	4	7	
*Klebsiella oxytoca*	10 (6.2)	9	1	
*Enterobacter cloacae* complex	14 (8.6)	13	1	
*Citrobacter freundii*	2 (1.3)	0	2	
*Morganella morganii*	3 (1.8)	3	0	
*Proteus mirabilis*	3 (1.8)	2	1	
*Enterobacter aerogenes*	2 (1.3)	1	1	
*Hafnia alvei*	1 (0.6)	1	0	
Gram-negative aerobic and anaerobic bacteria	*13 (8.1)*	*11 (11)*	*2 (0.3)*	*0.13*
*Pseudomonas aeruginosa*	10 (6.2)	9	1	
*Acinetobacter baumannii*	1 (0.6)	1		
*Stenotrophomonas maltophilia*	2 (1.3)	1	1	
Gram-negative anaerobic bacteria	*23 (14.2)*	*8 (8)*	*15 (23)*	*0.03*
*Bacteroides fragilis*	10 (6.2)	2	8	
*Prevotella* spp.	10 (6.2)	5	5	
Other anaerobes	3 (1.8)	1	2	
Gram-positive bacteria	*38 (23.4)*	*22 (23)*	*14 (21)*	*0.85*
*Enterococcus faecalis*	18 (11.1)	9	7	
Enterococcus faecium	4 (2.4)	3	1	
*Streptococcus*	6 (3.7)	4	2	
*Staphylococcus aureus*	7 (4.4)	5	2	
*Staphylococcus epidermidis*	3 (1.8)	1	2	
Fungi				
Candida	*6 (3.7)*	*5 (0.5)*	*1 (0.2)*	*0.4*
*C. albicans*	4 (2.4)	3	1	
*C. glabrata*	2 (1.3)	2	0	

Italic values indicate significance of *P* value (*P* < 0.05)

### Bacteria recovered in pancreatic samples (Table 3)

Of the 107 positive samples, 36 (33.4%) contained more than one bacterial species. In all, 162 different species were identified. Aerobic bacteria accounted for 82% of the species recovered, anaerobic bacteria for 14%, and *Candida* for 4%. *Escherichia coli* was the most common microorganism recovered from pancreatic samples (Table 3). The distribution of bacterial species was similar in the exposed and unexposed groups, except for *Enterobacter cloacae* complex which was more frequent in the exposed group (*p *= 0.02), as were Gram-negative anaerobic bacteria (*p *= 0.03). Of 18 patients with positive blood cultures, only 3 (16%) were polymicrobial, with the same bacterial species as in the pancreatic samples. Of the remaining 15 patients with monomicrobial blood culture, 10 had the same bacterial specie as in the monomicrobial pancreatic samples and 5 had only one of the bacterial species recovered in the polymicrobial pancreatic samples. Of the 156 bacterial species recovered from the 106 positive samples, 34 (22%) were MDRB and 17 (11%) were XDRB, with no significant difference between the exposed and unexposed groups (Additional file 2). Of the 56 patients, 25 (45%) developed a drug-resistant pancreatic infection including 15 with an MDRB and 10 with an XDRB. The 10 patients with an XDRB infection had a mean of 4.6 ± 2 pancreatic procedures and consistently required surgical necrosectomy. These 10 patients were all exposed to prolonged broad-spectrum antibiotic therapy before sampling. Among them, 5 kept the same bacterial species in serial samples, with the acquisition of resistances over time. Adjusting the antibiotic regimen to the susceptibility test results failed to sterilize the pancreatic samples, even after several weeks (Additional file 3). Of the 56 patients, 4 (7%) had pancreatic cultures positive for *Candida* at some point, including one who also had a blood culture positive for *Candida*.

### Patient outcomes in the exposed and unexposed groups

No significant differences were demonstrated between the two groups for the main complications, ICU stay length, ICU mortality, or hospital mortality.

## Discussion

The main finding from this retrospective cohort study is that the microbiology of pancreatic samples from critically ill patients with suspected IPN was not sterilized in patients on antibiotics for more than 24 h at sample collection. Thus, no significant differences were found between the exposed and unexposed groups regarding the proportion of positive samples, or identification of MDRB or XDRB, whereas *Enterobacter cloacae* complex and Gram-negative anaerobic bacteria were more frequent in the exposed group. Also, complications and mortality were similar in the two groups. Finally, the patient characteristics before the first sample collection were not significantly different between the exposed and unexposed groups.

Organ failure is common in patients with IPN [34]. The recommendation when following the step-up strategy is to wait at least 4 weeks if possible before performing a pancreatic procedure. During this period, antibiotics are often given due to sepsis or septic shock or to a documented infection outside the pancreas (e.g., nosocomial pneumonia, catheter-related infection, or bacteraemia). Negative samples collected while the patient was receiving antibiotics may indicate absence of an infection, presence of an infection that is responding to antibiotics, or a false-negative result. In our study, most of the patients with suspected IPN had positive cultures, with no difference between the exposed and unexposed groups. Thus, pancreatic sample cultures seem to preserve their diagnostic value even in patients on antibiotics and, in addition, provide valuable antimicrobial susceptibility information. If the cultures are negative, the diagnosis of IPN should be reappraised. Moreover, as in previous studies [9, 10], our results showed a high proportion of patients (86%) with a confirmed IPN by positive sample culture collected during drainage procedure or necrosectomy. These findings are consistent with international recommendations [4, 7, 8, 15, 16] advocating against the routine use of fine needle aspiration to confirm IPN.

In a study from England and Wales, antibiotics were given before the first pancreatic sample was collected in 439/712 (62%) patients [35], compared to 59% in our study. However, the inclusion criterion was acute pancreatitis, as opposed to suspected IPN in our study, and the patients were not all critically ill, with only 60/439 having sepsis. Furthermore, IPN was the reason for the first, second, and third courses of antibiotics in only 8, 0, and 1 patients, respectively, whereas in our study, a third of the patients had septic shock. Piperacillin/tazobactam was the most common antimicrobial used (34%), similar to our cohort (30%). In a retrospective study of a prospective database in the US of 182 consecutive symptomatic patients who had undergone pancreatic procedures for walled-off necrosis, 41% had culture-proven IPN [36]. Of these patients with positive cultures, 70% received antibiotics within 14 days before sample collection; and of the patients exposed to antibiotics before first sample collection, 70% had proven IPN. These results are consistent with ours, as are reports that about a third of samples contain more than one identified organism and that Gram-negative bacteria predominate [27, 37–39].

About a third of our patients had MDRB or XDRB identified in their pancreatic samples. Highly resistant strains of several bacterial species are becoming increasingly common in ICU patients [31]. In addition, among patients with acute pancreatitis, 50% to 85% have been found to have highly resistant bacteria [27, 28, 39]. Infections due to MDRB and XDRB are associated with considerably higher morbidity, mortality, and healthcare costs compared to those due to less resistant bacteria [39]. Acute pancreatitis with IPN runs a prolonged course that is often punctuated by a series of complications. Thus, many patients have long ICU and hospital stays associated with a high risk of cross-transmission [29], receive multiple courses of antibiotics, and require more than one pancreatic procedure. After a drainage procedure, more than half the patients require necrosectomy [9, 10]. The median number of procedures per patient in our study was 2. This complicated course may increase the opportunities for developing highly resistant bacteria.

*Candida* was identified in only 7% of our patients. In previous studies, this proportion varied widely, from 5% to 68.5%, perhaps in part due to differences in patient selection criteria. Prophylactic antibiotic therapy increases the risk of fungal infections which are associated with increased morbidity and mortality rates [40–42] and is not recommended in international guidelines. The absence of prophylactic antibiotic therapy in our patients may explain the low frequency of fungal infections.

In our study, antibiotic therapy given before sample collection failed to sterilize the pancreatic samples, even when broad-spectrum agents were given for prolonged periods. One possible explanation is poor penetration of the antibiotics into the fluid collections and necrotic foci. Very few data are available on this point. In two studies, after a single intravenous dose of ertapenem or imipenem, concentrations in pancreatic tissue and juice, respectively, were low but above the minimum inhibitory concentrations for the main pathogens responsible for intra-abdominal infections [22, 43, 44]. Several studies of animal models of acute pancreatitis [24–26] showed penetration within necrotic tissue to levels above the minimal inhibitory concentrations of common pathogens, and a single study [23] demonstrated good cefepime concentrations in pancreatic pseudocyst fluid or pancreatic resection specimens for cancer. However, pancreatic necrotic tissue during acute pancreatitis was not studied. If penetration is poor in necrotic tissue, then the sharp rise in highly resistant bacteria among patients with acute pancreatitis supports the use of antibiotic-sparing strategies in patients with IPN [37]. Antibiotic de-escalation based on antimicrobial susceptibility test results is needed to reduce the development of resistance [29, 30]. The cornerstone of IPN management is the removal of infected fluids and necrotic tissues according to the step-up strategy. Systemic antibiotics alone are not recommended for IPN [4, 5, 15, 16].

Blood cultures were positive in about a third of our patients, in keeping with earlier findings [9]. Antibiotic therapy before blood sample collection from patients with severe sepsis has been reported to markedly decrease the likelihood of positive blood cultures, even when time from antibiotic initiation to sampling was short [45]. Thus, obtaining pancreatic samples is crucial in patients with suspected IPN. For patients in septic shock (about one-third in our study), antibiotics must be started immediately. For the other patients, the question is whether to withhold antibiotics until the causative agent is identified or to start antibiotics then discontinue them promptly if the microbiological tests are negative.

Moreover, the low frequency of fungal infections in our study suggests that antifungal agents may be appropriate only in patients with a documented fungal infection, and in those with septic shock provided the treatment is stopped if the samples are negative for fungi.

Regarding the recovered microorganisms, the exposed group had more *Enterobacter cloacae* complex and non-fermenting Gram-negative bacteria and the unexposed group more Gram-negative anaerobic bacteria. *Candida* also seemed more frequent in the exposed group (*n* = 5 versus *n* = 1) but the difference was not significant, probably due to the small sample size. These disparities in microbial findings can be explained by exposure to antibiotics, which had a median duration of 7 days before bacteriological sampling in the exposed group. Other patient outcomes in our study were comparable in the exposed and unexposed groups, except for a higher overall number of antibiotics in the exposed group. Similarly, in a 2019 retrospective cohort study of ICU patients with acute pancreatitis, mortality was not different between the groups with vs. without antibiotic therapy at ICU admission [46].

The main limitation of our study is the retrospective single center design, with no standardization of the antibiotic treatment strategy. No prophylactic antibiotics were used in any patients. However, we did not have information on antibiotic exposure before ICU admission, on the reason for the timing of interventions, or on the reasons for discontinuing antibiotic therapy. Few guidelines exist about when to start and stop antibiotics or which antibiotics to use. Empirical therapy with antibiotics active on gastrointestinal organisms followed by adjustments based on antimicrobial susceptibility tests has been suggested [1, 7, 16]. We also had no information on nosocomial infections at extra-abdominal sites. In patients with multiple pancreatic fluid collections and/or necrotic foci, samples were not routinely obtained from all foci. This fact may have resulted in false-negative culture findings. Furthermore, patients categorized in the unexposed group may have received previous antimicrobial therapy for more than 24 h that may have affected the microbiological findings. However, given that cultures were positive in 86% of both patients exposed and unexposed to antibiotics within 24 h before sampling, in keeping with earlier data [9, 10], we are confident that previous antimicrobial therapy exposure is unlikely to have sterilized the samples. No information was available on intestinal carriage or known colonization of these patients, or on the mechanisms of acquired antibiotic resistance in the patients with highly resistant strains. Data on inflammatory status including body temperature, leukocytosis, and procalcitonin levels were also lacking, as they were not reliably recorded in the medical files. However, these data are of limited relevance in critically ill patients. Despite these limitations, our study provides the first evidence on the impact of prior antibiotics on pancreatic sample microbiology in patients managed with the currently recommended step-up strategy for suspected IPN and no prophylactic antibiotics. We collected a vast amount of information on the types and timing of antibiotic treatments, culture results, and antimicrobial susceptibilities.

## Conclusion

In patients admitted to the ICU with suspected IPN, starting antibiotic therapy more than 24 h before collection of the first pancreatic sample did not seem to sterilize microbiological cultures and had a limited impact on microbiological findings. The cultures were positive in most patients. It therefore seems appropriate to start empirical antibiotics immediately in patients with sepsis, septic shock, or a new organ failure. A pancreatic sample should then be obtained as soon as possible to identify the organism and assess the susceptibility profile. Given the high prevalence of bacteria exhibiting resistance to many agents, in other situations, antibiotic therapy may be best reserved for those patients with positive pancreatic samples may be appropriate, in order to limit the unnecessary use of antibiotics.

## **Supplementary information**

**Additional file 1: Table S1**. Modified Marshall scoring system for organ dysfunction.

**Additional file 2: Table S2.** Microbiological culture results in 23 patients with infected pancreatic necrosis and multidrug-resistant (MDR) or extensively drug-resistant (XDR) bacteria in pancreatic samples.

**Additional file 3: Table S3.** Details of antibiotic therapy and microbiological results of pancreatic samples in 10 patients with extensively drug-resistant bacteria during their hospital stays for infected pancreatic necrosis.

## Data Availability

The datasets used and analyzed during the current study are available from the corresponding author on reasonable request.

## Abbreviations

ICU: Intensive care unit
IPN: Infected pancreatic necrosis
MDRB: Multidrug-resistant bacteria
XDRB: Extensively drug-resistant bacteria
