# Supplementary material for: Impact of prior antibiotics on infected pancreatic necrosis microbiology in ICU patients: a retrospective cohort study
Source: Ann Intensive Care. 2020 Jun 15;10:82. doi: 10.1186/s13613-020-00698-0 (PMC7295875; doi:10.1186/s13613-020-00698-0)
Supplement: Supplementary file 1 — Additional file 1: Table S1. Modified Marshall scoring system for organ dysfunction. [file 13613_2020_698_MOESM1_ESM.docx]

**ADDITIONAL FILES**

|  | Score | | | | |
| --- | --- | --- | --- | --- | --- |
| Organ system | 0 | 1 | 2 | 3 | 4 |
| Respiratory (PaO_2_/FiO_2_) | >400 | 301-400 | 201-300 | 101-200 | ≤101 |
| Renal^a^: serum creatinine, μmol/L | ≤134 | 134-169 | 170-310 | 311-439 | >439 |
| Cardiovascular: systolic blood pressure, mm Hg^b^ | >90 | <90, fluid responsive | <90, not fluid responsive | <90, pH <7.3 | <90, pH <7.2 |
| For non-ventilated patients, the FiO_2_ can be estimated as follows: | | | | | |
| Supplemental oxygen (L/min) | FiO_2_ (%) |  |  |  |  |
| Room air | 21% |  |  |  |  |
| 2 | 25% |  |  |  |  |
| 4 | 30% |  |  |  |  |
| 6-8 | 40% |  |  |  |  |
| 9-10 | 50% |  |  |  |  |

**Additional file 1: Table S1. Modified Marshall scoring system for organ dysfunction**

A score of 2 or more in any of the three organ systems defines the presence of organ failure.

^a^The score for patients with preexisting chronic renal failure depends on the extent of further deterioration of baseline renal function. No formal correction exists for a baseline serum creatinine ≥134 μmol/L or ≥1.4 mg/dl.

^b^Off inotropic support
