# Supplementary material for: Impact of prior antibiotics on infected pancreatic necrosis microbiology in ICU patients: a retrospective cohort study
Source: Ann Intensive Care. 2020 Jun 15;10:82. doi: 10.1186/s13613-020-00698-0 (PMC7295875; doi:10.1186/s13613-020-00698-0)
Supplement: Supplementary file 2 — Additional file 2: Table S2. Microbiological culture results in 23 patients with infected pancreatic necrosis and multidrug-resistant (MDR) or extensively drug-resistant (XDR) bacteria in pancreatic samples. [file 13613_2020_698_MOESM2_ESM.docx]

**Additional file 2: Table S2. Microbiological culture results in 23 patients with infected pancreatic necrosis and multidrug-resistant (MDR) or extensively drug-resistant (XDR) bacteria in pancreatic samples**

| **Bacteria** | Total patients with MDR  N=13  MDR bacterial species  n=43 (100%) | Exposed patients  N=8  MDR bacterial Species  n=34 (79%) | Unexposed patients  N=6  MDR bacterial species  n=9 (21%) | Total patients with XDR  N=10  XDR bacterial  species  n=17 (100%) | Exposed patients  N=6  XDR bacterial  species  n=8 (47%) | Unexposed patients  N=4  XDR bacterial  species  n=9 (53%) |
| --- | --- | --- | --- | --- | --- | --- |
| **Gram-negative Enterobacteriaceae** | **30 (70%)** | **23 (54%)** | **7 (16%)** | **14 (82%)** | **5 (30%)** | **9 (52%)** |
| *Escherichia coli* | 14 | 8 | 6 |  |  | 1 |
| *Klebsiella pneumoniae* |  |  |  |  | 3 | 4 |
| *Klebsiella oxytoca* | 4 | 4 |  |  |  |  |
| *Enterobacter cloacae* complex | 8 | 8 |  |  | 2 | 4 |
| *Citrobacter freundii* | 1 |  | 1 |  |  |  |
| *Morganella morganii* | 3 | 3 |  |  |  |  |
| **Gram-negative aerobic and anaerobic bacteria** | **4 (9.5%)** | **3 (7%)** | **1 (2.5%)** | **3 (18%)** | **3 (18%)** | **0** |
| *Pseudomonas aeruginosa* |  | 3 |  |  | 3 |  |
| *Stenotrophomonas maltophilia* |  |  | 1 |  |  |  |
| **Gram-negative anaerobic bacteria** | **5 (11%)** | **5 (11%)** | **0** |  |  |  |
| *Bacteroides fragilis* |  | 4 |  |  |  |  |
| *Prevotella* spp |  | 1 |  |  |  |  |
| **Gram-positive bacteria** | **4 (9.5%)** | **3 (7%)** | **1 (2.5%)** |  |  |  |
| *Enterococcus faecalis* |  | 1 |  |  |  |  |
| *Enterococcus faecium* |  | 2 | 1 |  |  |  |
