# Supplementary material for: Impact of prior antibiotics on infected pancreatic necrosis microbiology in ICU patients: a retrospective cohort study
Source: Ann Intensive Care. 2020 Jun 15;10:82. doi: 10.1186/s13613-020-00698-0 (PMC7295875; doi:10.1186/s13613-020-00698-0)
Supplement: Supplementary file 3 — Additional file 3: Table S3. Details of antibiotic therapy and microbiological results of pancreatic samples in 10 patients with extensively drug-resistant bacteria during their hospital stays for infected pancreatic necrosis. [file 13613_2020_698_MOESM3_ESM.docx]

**Additional file 3: Table S3. Details of antibiotic therapy and microbiological results of pancreatic samples in 10 patients with extensively drug-resistant bacteria during their hospital stays for infected pancreatic necrosis**

|  | **Weeks** | **1** | **2** | **3** | **4** | **5** | **6** | **7** | **8** | **9** | **10** | **11** | **12** | **13** | **14** | **15** | **16** |
| --- | --- | --- | --- | --- | --- | --- | --- | --- | --- | --- | --- | --- | --- | --- | --- | --- | --- |
| Patient n°1 | Cephalosporin | x |  |  |  |  |  |  |  |  |  |  |  |  |  |  |  |
|  | Penems |  |  |  | x | x | x | x | x | x | x | x | x | x |  | x | x |
|  | **Bacterial agent n°1** | ***0*** |  |  |  |  | ***ECC***  ********* |  |  | ***ECC***  ********* |  |  | ***ECC***  ********* |  |  | ***ECC***  ********* |  |
| Patient n°2 | Penicillin |  | x | x |  |  |  |  |  |  |  |  |  |  |  |  |  |
|  | Pip-Tazobactam |  |  |  |  |  | x |  |  |  |  |  |  |  |  |  |  |
|  | Penems |  |  |  | x |  |  | x | x |  |  |  |  |  |  |  |  |
|  | **Bacterial agent n°1** |  | ***E.coli***  ******* |  |  |  |  | ***E.coli***  ********* |  |  |  |  |  |  |  |  |  |
|  | **Bacterial agent n°2** |  |  |  |  |  |  | ***Strept**** |  |  |  |  |  |  |  |  |  |
| Patient n°3 | Other |  |  |  |  |  | x | x |  |  |  |  |  |  |  |  |  |
|  | Colistin |  |  |  |  |  |  |  |  | x | x |  |  |  |  |  |  |
|  | Fluoroquinolone |  |  |  |  |  |  |  |  | x | x |  |  |  |  |  |  |
|  | Cephalosporin |  |  |  |  |  |  |  |  | x | x |  |  |  |  |  |  |
|  | Penicillin |  | x |  |  |  | x | x |  |  |  |  |  |  |  |  |  |
|  | Pip-Tazobactam |  |  |  |  | x |  |  | x |  |  |  |  |  |  |  |  |
|  | **Bacterial agent n°1** |  |  |  | ***0*** | ***ECC***  ******** |  |  |  | ***ECC***  ******** |  |  |  |  |  |  |  |
|  | **Bacterial agent n°2** |  |  |  |  | ***Bact* sp**  ***** |  |  |  | ***Pyo***  ******** | ***Pyo***  ********* |  |  |  |  |  |  |
|  | **Bacterial agent n°3** |  |  |  |  | ***E. fae-cium***  ******* |  |  |  | ***Pro-teus***  ******* |  |  |  |  |  |  |  |
| Patient n°4 | Other |  |  |  |  |  |  |  | x | x | x | x | x |  |  |  |  |
|  | Colistin |  |  |  |  |  |  |  | x | x | x | x | x |  |  |  |  |
|  | Penicillin |  |  |  |  |  |  |  | x | x | x | x | x |  |  |  |  |
|  | Penems |  |  | x | x |  |  | x |  |  |  |  |  |  |  |  |  |
|  | **Bacterial agent n°1** |  |  |  |  |  |  |  | ***Pyo***  ********* | ***Pyo***  ********* |  |  |  |  |  |  |  |
|  | **Bacterial agent n°2** |  |  |  |  |  |  |  | ***E. fae-calis***  ******* |  |  |  |  |  |  |  |  |
| Patient n°5 | Other |  |  |  |  |  |  |  |  |  |  |  | x | x | x | x | x |
|  | Pip-Tazobactam |  |  |  |  |  |  |  |  |  |  |  | x | x | x | x | x |
|  | Cephalosporin | x | x | x | x |  |  |  |  |  |  |  |  |  |  |  |  |
|  | Penems |  |  |  |  |  | x | x | x | x | x |  |  |  |  |  |  |
|  | **Bacterial agent n°1** |  |  |  |  |  | ***KP***  ********* |  | ***KP***  ********* | ***KP***  ********* |  |  |  |  |  |  |  |
|  | **Bacterial agent n°2** |  |  |  |  |  | ***Prevo**** |  | ***E. coli**** |  |  |  | ***E. coli**** |  |  |  | ***E. coli***** |
|  | **Bacterial agent n°3** |  |  |  |  |  | ***Bact* sp**  ***** |  |  |  |  |  | ***Bact* sp**  ***** |  |  |  | ***Bact* sp**  ***** |
|  | **Bacterial agent n°4** |  |  |  |  |  |  |  |  |  |  |  |  |  |  |  | ***E. fae-cium***  ******** |
| Patient n°6 | Pip-Tazobactam |  |  |  |  | x |  |  |  |  |  |  |  |  |  |  |  |
|  | Cephalosporin | x | x | x | x |  |  |  |  |  |  |  |  |  |  |  |  |
|  | Penems |  |  |  |  |  | x | x | x |  |  |  |  |  |  |  |  |
|  | **Bacterial agent n°1** |  |  |  | ***KP***  ******* | ***KP***  ******* |  | ***KP***  ******* |  |  |  |  |  |  |  |  |  |
|  | **Bacterial agent n°2** |  |  |  |  |  | ***ECC***  ******** | ***ECC***  ********* |  |  |  |  |  |  |  |  |  |
| Patient n°7 | Other |  |  |  |  |  |  |  |  | x | x | x | x |  |  |  |  |
|  | Penicillin | x | x |  | x | x | x | x |  |  |  | x |  |  |  |  |  |
|  | Pip-Tazobactam |  |  | x |  |  |  |  |  |  |  |  |  |  |  |  |  |
|  | Penems |  |  |  |  |  |  |  |  | x | x | x | x | x |  |  |  |
|  | **Bacterial agent n°1** | ***0*** | ***0*** |  | ***E. coli**** |  |  |  |  |  | ***E. coli***** |  |  |  |  |  |  |
|  | **Bacterial agent n°2** |  |  |  | ***E. fae-cium***  ******* |  |  |  |  |  | ***E. fae-cium***  ******** |  |  |  |  |  |  |
|  | **Bacterial agent n°3** |  |  |  |  |  |  |  |  |  |  | ***KP***  ********* |  |  |  |  |  |
| Patient n°8 | Other |  |  |  |  |  |  |  |  | x | x | x | x |  |  |  |  |
|  | Penicillin |  |  |  |  |  |  |  |  |  |  | x |  |  |  |  |  |
|  | Pip-Tazobactam | x |  |  |  |  |  |  |  |  |  |  |  |  |  |  |  |
|  | Penems |  |  |  |  |  |  |  | x | x | x | x | x | x | x | x |  |
|  | **Bacterial agent n°1** |  | ***0*** |  |  |  |  |  | ***Pyo***  ******* |  |  |  |  | ***Pyo***  ******** |  |  |  |
|  | **Bacterial agent n°2** |  |  |  |  |  |  |  | ***Steno**** |  |  |  |  | ***KP***  ********* |  |  |  |
|  | **Bacterial agent n°3** |  |  |  |  |  |  |  |  |  |  | ***E. fae-cium***  ******** |  |  |  |  |  |
| Patient n°9 | Cephalosporin |  | x |  |  |  |  |  |  |  |  |  |  |  |  |  |  |
|  | Penems |  |  | x | x |  |  |  |  |  |  |  |  |  |  |  |  |
|  | **Bacterial agent n°1** |  |  | ***ECC***  ******** | ***ECC***  ********* |  |  |  |  |  |  |  |  |  |  |  |  |
|  | **Bacterial agent n°2** |  |  |  | ***E. fae-calis***  ******* |  |  |  |  |  |  |  |  |  |  |  |  |
| Patient n°10 | Other |  | x | x | x | x |  |  |  |  |  |  |  |  |  |  |  |
|  | Penems |  | x | x | x | x |  |  |  |  |  |  |  |  |  |  |  |
|  | **Bacterial agent n°1** |  |  | ***KP***  ******** | ***KP***  ******** | ***KP***  ********* |  |  |  |  |  |  |  |  |  |  |  |

0 : sterile culture

* No drug-resistant bacteria

** Multidrug-resistant bacteria

*** Extensively drug-resistant bacteria

Other: fluoroquinolone, macrolide, linezolid, vancomycin

*ECC: Enterobacter cloacae* complex

*E.coli: Escherichia coli*

*Strept: Streptococcus pneumoniae*

*Bact sp: Bacteroides sp*

*Pyo: Pseudomonas aeruginosa*

*E. faecium: Enterococcus faecium*

*E. faecalis: Enterococcus faecalis*

*KP: Klebsiella pneumoniae*

*Prevo: Prevotella sp*

*Steno: Stenotrophomonas maltophilia*
